# The synthetic triterpenoid RTA dh404 (CDDO-dhTFEA) restores endothelial function impaired by reduced Nrf2 activity in chronic kidney disease^☆^

**DOI:** 10.1016/j.redox.2013.10.007

**Published:** 2013-10-31

**Authors:** Mohammad A. Aminzadeh, Scott A. Reisman, Nosratola D. Vaziri, Stan Shelkovnikov, Seyed H. Farzaneh, Mahyar Khazaeli, Colin J. Meyer

**Affiliations:** a Division of Nephrology and Hypertension, Schools of Medicine and Biological Science, University of California-Irvine, 101 The City Drive, City Tower, Suite 400, Orange, CA 92868, USA; b Reata Pharmaceuticals, Inc., 2801 Gateway Dr. Ste 150, Irving, TX 75063, USA

**Keywords:** 12-LO, 12-lipoxygenase, AT_1_, angiotensin II receptor type 1, CDDO-dhTFEA, CDDO-9,11-dihydro-trifluoroethyl amide, CKD, chronic kidney disease, GAPDH, glyceraldehyde 3-phosphate dehydrogenase, Ho-1, heme oxygenase-1, IKKβ, IkappB kinase β, Keap1, Kelch like ECH-associated protein 1 MCP-1, monocyte chemoattractant protein-1, NAD(P)H, nicotinamide adenine dinucleotide (phosphate), reduced form, NF-κB, nuclear factor *κ-*light chain*-*enhancer of activated B cells, NO, nitric oxide, NOS, nitric oxide synthase, Nrf2, nuclear factor erythroid 2-related factor 2, NT, nitrotyrosine, PhE, phenylephrine, Rac1, Ras-related C3 botulinum toxin substrate 1, ROS, reactive oxygen species, Sod2, superoxide dismutase 2, Nrf2, Chronic kidney disease, Oxidative stress, Inflammation, Aorta, Synthetic triterpenoid, Bardoxolone methyl

## Abstract

Chronic kidney disease (CKD) is associated with endothelial dysfunction and accelerated cardiovascular disease, which are largely driven by systemic oxidative stress and inflammation. Oxidative stress and inflammation in CKD are associated with and, in part, due to impaired activity of the cytoprotective transcription factor Nrf2. RTA dh404 is a synthetic oleanane triterpenoid compound which potently activates Nrf2 and inhibits the pro-inflammatory transcription factor NF-κB. This study was designed to test the effects of RTA dh404 on endothelial function, inflammation, and the Nrf2-mediated antioxidative system in the aorta of rats with CKD induced by 5/6 nephrectomy. Sham-operated rats served as controls. Subgroups of CKD rats were treated orally with RTA dh404 (2 mg/kg/day) or vehicle for 12 weeks. The aortic rings from untreated CKD rats exhibited a significant reduction in the acetylcholine-induced relaxation response which was restored by RTA dh404 administration. Impaired endothelial function in the untreated CKD rats was accompanied by significant reduction of Nrf2 activity (nuclear translocation) and expression of its cytoprotective target genes, as well as accumulation of nitrotyrosine and upregulation of NAD(P)H oxidases, 12-lipoxygenase, MCP-1, and angiotensin II receptors in the aorta. These abnormalities were ameliorated by RTA dh404 administration, as demonstrated by the full or partial restoration of the expression of all the above analytes to sham control levels. Collectively, the data demonstrate that endothelial dysfunction in rats with CKD induced by 5/6 nephrectomy is associated with impaired Nrf2 activity in arterial tissue, which can be reversed with long term administration of RTA dh404.

## Highlights

• Endothelial dysfunction in rats with chronic kidney disease is associated with impaired aortic Nrf2 activity.
• Long-term administration of the Nrf2 activator RTA dh404 ameliorated downregulation of Nrf2 and vascular dysfunction induced by chronic kidney disease.
• Long-term administration of the Nrf2 activator RTA dh404 ameliorated chronic kidney disease-induced accumulation of nitrotyrosine and upregulation of NF-κB target genes.

## Graphical abstract

**Figure:**
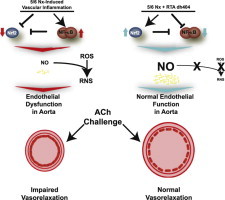

## Introduction

Chronic kidney disease (CKD) is associated with accelerated cardiovascular disease, the major cause of mortality in this patient population [1]. A major contributor to cardiovascular disease in CKD is endothelial dysfunction caused by vascular oxidative stress, inflammation, and dysregulation of key pathways, such as the renin-angiotensin aldosterone axes [2]. Endothelial dysfunction in CKD is primarily driven by oxidative stress, which limits bioavailability of nitric oxide (NO) by lowering its production and promoting its inactivation [3]. Oxidative stress reduces NO bioavailability through the reaction of reactive oxygen species (ROS) with NO to generate reactive nitrogen species, namely peroxynitrite [2]. Interventions aimed at alleviation of oxidative stress can reverse endothelial dysfunction and protect against cardiovascular disease. For example, the addition of functional catalase attenuates the oxidative stress-mediated decrease in the contractile response of vascular tissue from 5/6 nephrectomized rats [4]. Further, the superoxide dismutase mimetic tempol reduces blood pressure and oxidative stress and restores the normal contractile responsiveness of aorta from pre-eclamptic rats [5].

Under physiological conditions, the potentially deleterious effects of ROS are counterbalanced by a system of enzymatic and non-enzymatic antioxidants, which are transcriptionally regulated by nuclear factor erythroid 2-related factor 2 (Nrf2) [6]. Activation of Nrf2 involves its translocation to the nucleus, where it binds to antioxidant response elements in the promoter region of its target genes, leading to induction of many antioxidant and cytoprotective enzymes and related proteins [7,8]. Target genes of Nrf2 include, but are not limited to, superoxide dismutase 2 (Sod2) [9] and heme oxygenase-1 (Ho-1) [10]. Sod2 facilitates detoxification of superoxide radicals and is subcellularly located in the mitochondria, whereas Ho-1 catalyzes the breakdown of heme into the antioxidant molecule biliverdin and the anti-inflammatory molecule carbon monoxide. However, under pathological conditions and periods of chronic inflammation, Nrf2 activity and expression of its cytoprotective target genes can be profoundly decreased, often contributing to progression of disease [11]. Moreover, pharmacological activation of Nrf2 has been shown to be critical for protection of endothelial cells under conditions of oxidative stress. For example, resveratrol, a phytochemical with weak Nrf2 activating properties, protects wild-type mouse endothelial function, as evaluated by assessing acetylcholine-induced vasodilation, but these salutary effects were diminished in Nrf2-null mice [12]. Further, another phytochemical with Nrf2 activating properties, namely epicatechin, reduces hypertension and endothelial dysfunction in the deoxycorticosterone acetate-salt rat model [13].

RTA dh404 and its analogs are oleanolic acid-derived synthetic triterpenoid compounds which potently activate the Nrf2-Kelch like ECH-associated protein 1 (Keap1) pathway [14,15]. Synthetic oleanane triterpenoids are also potent inhibitors of the NF-κB inflammatory pathway through both direct (*i.e.*, inhibition of IKKβ kinase activity) and indirect mechanisms (*i.e.*, detoxification of reactive oxygen species) [16]. The effects of synthetic oleanane triterpenoids on activation of the antioxidant Nrf2 pathway and inhibition of the pro-inflammatory NF-κB pathway are consistent with the notion that oxidative stress and inflammation are inextricably linked and form a self-perpetuating cycle that is paramount to the pathogenesis of many diseases [3].

Recently, it was demonstrated that long term (12 weeks) administration of RTA dh404 resulted in attenuation of oxidative stress, inflammation, and fibrosis in the remnant kidneys of rats with CKD induced by 5/6 nephrectomy, which was accompanied by a reduction in proteinuria and mean arterial pressure; induction or rescue of Nrf2 and its target genes, and decreased activation and expression of NF-κB and its target genes in kidney tissue were also observed (Aminzadeh et al., [11], *Xenobiotica*, *in press*). However, RTA dh404 or other analogs have not yet been tested for their effects on CKD-induced vascular disease. Therefore, this study was performed to investigate the effects of CKD and long term treatment with the potent Nrf2 activator, RTA dh404, on endothelial function and on Nrf2 and oxidative and inflammatory pathways in the arterial tissue of 5/6 nephrectomized rats.

## Methods

### Materials

All reagents were purchased from Sigma Aldrich (St. Louis, MO). RTA dh404 was synthesized and provided by Reata Pharmaceuticals, Inc. (Irving, TX). The chemical name for RTA dh404 is CDDO-9,11-dihydro-trifluoroethyl amide (CDDO-dhTFEA).

### Animals

Male Sprague-Dawley rats were randomly assigned to the CKD or normal control groups. The rats assigned to the CKD group were subjected to 5/6 nephrectomy by surgical resection using dorsal incisions, as described previously [17], while those animals assigned to the control group were subjected to sham operation (*n*=6). Rats with CKD were orally administered RTA dh404 (2 mg/kg) or vehicle (sesame oil) once daily for 12 weeks (*n*=9/group) starting immediately prior to surgery. The thoracic aorta was prepared and tension experiments performed as previously described [18].

### Western blot analyses

Cytoplasmic and nuclear extracts were prepared, and western blots were performed as previously described [19]. Target proteins in the cytoplasmic and nuclear fractions of the aortic tissue were evaluated by western blot analysis using commercially available antibodies. Antibodies against Nrf2, Keap1, Ho-1, AT_1_ (Santa Cruz Biotechnology, Santa Cruz, CA), nitrotyrosine, 12-lipoxygenase (12-LO) (Abcam, Cambridge, MA), gp91^phox^, Rac1 (BD Bioscience, San Jose, CA), p47phox, MCP-1 (Sigma Aldrich St. Louis, MO), and Sod2 (Calbiochem, Billerica, MA) were purchased from the designated sources. Antibodies against histone H1 and glyceraldehyde 3-phosphate dehydrogenase (GAPDH) (Santa Cruz Biotechnology) were used for measurements of the housekeeping proteins for nuclear and cytosolic target proteins, respectively. The secondary antibodies for horseradish peroxidase-linked anti-sheep IgG and anti-goat IgG were purchase from Sigma Aldrich.

### Statistical analyses

Data were analyzed with Sigmaplot 12.0 (Systat, Inc., San Jose, CA) by one way-analysis of variance (ANOVA) followed by Duncan’s Multiple Range post-hoc test with significance set at *p*<0.05.

## Results

### Effect of RTA dh404 on acetylcholine-induced relaxation response in aorta from CKD rats

**Fig. 1:**
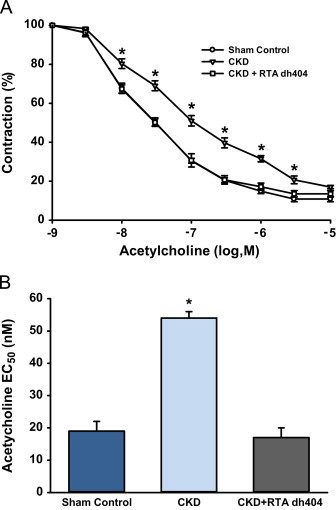
**Effect of RTA dh404 on acetylcholine-induced contractile response in aorta from CKD rats.** A. Cumulative concentration–response curves of acetylcholine in sham control, chronic kidney disease (CKD), or CKD+RTA dh404 rats are presented on a semi-log scale. The contraction obtained after phenylephrine is set to 100%. There were no differences in maximum contraction among groups (data not shown). B. EC_50_ values of acetylcholine concentrations calculated from concentration–response curves are presented. Values are presented as mean ± standard error of the mean. Asterisks indicate a statistically significant difference from both sham control and CKD+RTA dh404 groups (^⁎^*p*<0.05).

NO-dependent endothelial function was measured by vasodilation of aortic rings isolated from nephrectomized rats in response to acetylcholine after pre-contraction by phenylephrine. In confirmation of previous studies [20,21], acetylcholine-induced vasorelaxation was markedly impaired in the untreated CKD group. RTA dh404 administration normalized acetylcholine-induced vasorelaxation in the treated CKD rat group (Fig. 1A). The acetylcholine EC_50_ concentration was significantly increased almost 3-fold in the untreated CKD rats, and was restored to that observed in sham-operated controls by RTA dh404 administration (Fig. 1B). These data are consistent with the observation that RTA dh404 restores mean arterial pressure to sham control levels in the 5/6 nephrectomy model (Aminzadeh et al., [11], *submitted to Xenobiotica* ). RTA dh404 did not alter the maximum contraction evoked by PhE or KCl or the concentration–response of PhE, which are all decreased in rats with CKD (data not shown).

### Effect of RTA dh404 on Nrf2, Nrf2 target, and Keap1 protein expression in aorta from CKD rats

**Fig. 2:**
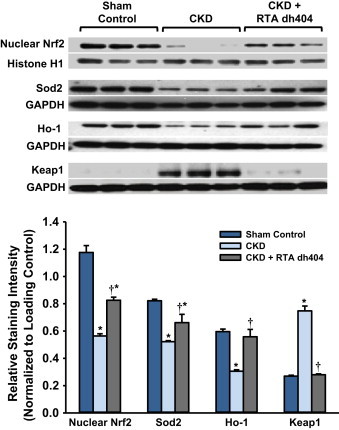
**Effect of RTA dh404 on Nrf2, Nrf2 target, and Keap1 protein expression in aorta from CKD rats**. Representative Western blots and group data are presented, depicting protein abundance of Nrf2, Nrf2 downstream gene products: superoxide dismutase 2 (Sod2) and heme oxygenase-1 (Ho-1), as well as Keap1 in the aortas of sham-operated control (*n*=6) and 5/6 nephrectomized rats [chronic renal failure (CKD)] treated with vehicle (CKD; *n*=9) or RTA dh404 (CKD+RTA dh404; *n*=9). Histone H1 served as the loading control for Nrf2, whereas GAPDH served as the loading control for Sod2, Ho-1, and Keap1. Asterisks indicate a statistically significant difference from sham control (^⁎^*p*<0.05). Daggers indicate a statistically significant difference from the CKD group (^†^*p*<0.05).

Aortic tissue was evaluated via immunoblotting for protein expression of Nrf2 in nuclear fractions (*i.e.*, activated Nrf2) and Ho-1, Sod2, and Keap1 in cytosolic fractions. CKD significantly lowered the Nrf2 content in the nucleus, as well as Ho-1 and Sod2 protein expression, whereas Keap1 protein expression was significantly increased (Fig. 2). RTA dh404 administration fully or partially restored the Nrf2 content in the nucleus, as well as Sod2, Ho-1, and Keap1 protein expression.

### Effect of RTA dh404 on NF-κB target protein expression in aorta from CKD rats

**Fig. 3:**
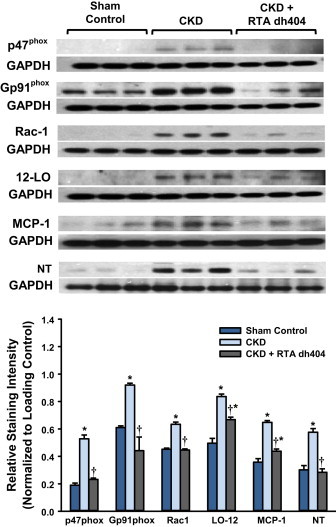
**Effect of RTA dh404 on NF-κB target protein expression in aorta from CKD rats**. Representative Western blots and group data are presented, depicting protein abundance of NAD(P)H oxidase subunits (p22^phox^, gp91^phox^, and Rac1), 12-lipoxygenase (12-LO), monocyte chemotactic protein-1 (MCP-1), and nitrotyrosine (NT) in the aortas from sham-operated control (*n*=6) and 5/6 nephrectomized rats [chronic kidney disease (CKD)] treated with vehicle (CKD; *n*=9) or RTA dh404 (CKD+RTA dh404; *n*=9). GAPDH served as the loading control. Asterisks indicate a statistically significant difference from sham control (^⁎^*p*<0.05). Daggers indicate a statistically significant difference from the CKD group (^†^*p*<0.05).

The untreated CKD rats exhibited significant increase in nitrotyrosine abundance and protein expressions of NAD(P)H oxidase subunits p47phox, Gp91^phox^, and Rac-1, as well as 12-lipoxygenase, and MCP-1. RTA dh404 administration fully or partially reversed these abnormalities (Fig. 3).

### Effect of RTA dh404 on angiotensin receptor protein expression in aorta from CKD rats

**Fig. 4:**
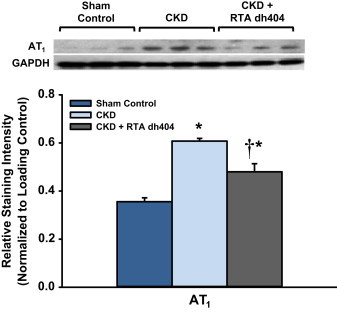
**Effect of RTA dh404 on angiotensin receptor protein expression in aorta from CKD rats**. Representative Western blots and group data depicting protein abundance of angiotensin receptor type 1 (AT_1_) in the aortas from sham-operated control (*n*=6) and 5/6 nephrectomized rats [chronic kidney disease (CKD)] treated with vehicle (CKD; *n*=9) or RTA dh404 (CKD+RTA dh404; *n*=9). GAPDH served as the loading control. Asterisks indicate a statistically significant difference from sham control (^⁎^*p*<0.05). Daggers indicate a statistically significant difference from the CKD group (^†^*p*<0.05).

The untreated CKD rats showed significantly increased protein expression of AT_1_ in the aortic tissue. RTA dh404 administration resulted in partial restoration of AT_1_ expression toward values found in the sham-operated control animals (Fig. 4).

## Discussion

As anticipated, the vehicle-treated CKD rats employed in the present study exhibited marked endothelial dysfunction as evidenced by impaired acetylcholine-induced vasodilation. Acetylcholine enhances NO production in endothelial cells, which facilitates vascular smooth muscle relaxation. Endothelial dysfunction in CKD is primarily due to oxidative stress which limits bioavailability of NO by several mechanisms, which include uncoupling of NO synthase (NOS) homodimers, depletion of NOS cofactor (tetrahydrobiopterin), accumulation of endogenous NOS inhibitor asymmetrical dimethyl arginine (ADMA), and inactivation of NO by superoxide [22]. In fact, endothelial dysfunction in the untreated CKD rats in this study was associated with significant upregulation of NAD(P)H oxidase, a major source of superoxide in the vascular tissue, and accumulation of nitrotyrosine, a major footprint of NO inactivation by superoxide. Administration of RTA dh404 resulted in the reversal of CKD-induced upregulation of NAD(P)H oxidase, which was accompanied by marked reduction in nitrotyrosine abundance. Moreover, RTA dh404 therapy restored expression of superoxide dismutase, a key enzyme for detoxification of superoxide. Thus, by reversing the upregulation of NAD(P)H oxidase and downregulation of Sod2, RTA dh404 administration likely results in decreased superoxide production. By attenuating oxidative stress and limiting superoxide level in the arterial wall, RTA dh404 treatment is hypothesized to increase NO availability, a phenomenon which is consistent with the observed improvement in endothelial function in the CKD animals.

Rats with CKD also displayed marked decreases in Nrf2 and its measured target proteins, as well as increases in biomarkers of inflammation and oxidative stress in the aorta. Activated Nrf2 (*i.e.*, Nrf2 in the nucleus) was decreased by over 50%, which was associated with decreases in both Sod2 and Ho-1 expression. Interestingly, Keap1, the repressor of Nrf2 and target of RTA dh404 was increased by CKD, suggesting that increased tethering of Nrf2 by Keap1 in the cytosol in rats with CKD contributes to oxidative stress in the aortic vasculature. Impaired Nrf2 activity in the untreated CKD rats was associated with upregulation of NAD(P)H oxidase subunits p47phox, Gp91^phox^, and Rac-1, which is often observed in concert with the increase in expression of AT_1_. Activation of AT_1_ receptor triggers NAD(P)H oxidase activation, increases superoxide production, events that can cause cellular damage and dysfunction in pathological conditions [23]. CKD also increased expression of 12-lipoxygenase which catalyzes oxidation of fatty acids to generate bioactive products capable of causing inflammation and NF-κB activation [24]. Further, 12-lipoxygenase produces oxidized phospholipids, which can modify the fluidity of the plasma membrane of cells and up-regulate expression of vascular endothelial growth factor (VEGF) [24,25]. VEGF, in turn, promotes angiogenesis by stimulating proliferation of endothelial cells and inducing vascular leakage and increased permeability, thereby enhancing disease progression [26]. Rats with CKD also had increased expression of the chemokine MCP-1 in the aorta. MCP-1 is implicated in the pathogenesis of vascular disease by promoting monocyte adhesion, infiltration, and transformation to macrophages and by stimulating smooth muscle cell migration and proliferation, among other inflammatory processes [27]. Finally, increased oxidative stress and inflammation were apparent, as indicated by increased nitrotyrosine abundance in aortic tissue from rats with CKD.

Administration of RTA dh404 to rats with CKD restored Nrf2 activity and expression of Sod2, and Ho-1, approaching values found in the sham-operated control rats. RTA dh404 also decreased Keap1, the repressor of Nrf2, to the level found in the control animals and prevented the development of the pro-inflammatory phenotype in aortic tissue, as manifested by decreases in MCP-1, NAD(P)H oxidase subunit expression, and 12-lipoxygenase. RTA dh404 administration was also associated with a decrease in the protein expression of AT_1_ receptors in aortic tissue from rats with CKD. While it is not known if the RTA dh404-associated decreased in AT_1_ receptor expression is direct or indirect, it does reflect a shift away from pro-inflammatory and vasoconstrictive phenotypes. All of the above discussed beneficial changes are consistent with the known Nrf2 activation and NF-κB inhibition properties of RTA dh404, which may at least, in part, contribute to the protection associated with RTA dh404 administration in this model of CKD.

In conclusion, endothelial dysfunction in rats with CKD was associated with upregulation of oxidative and inflammatory pathways, downregulation of endogenous antioxidant molecules, and impaired Nrf2 activity in the arterial tissue. Long-term administration of the potent Nrf2 activator RTA dh404 ameliorated CKD-induced Nrf2 dysfunction, oxidative stress, and inflammation in the aorta and restored endothelial function in 5/6 nephrectomized rats.
